# Supplementary material for: Dissecting the early steps of MLL induced leukaemogenic transformation using a mouse model of AML
Source: Nat Commun. 2020 Mar 16;11:1407. doi: 10.1038/s41467-020-15220-0 (PMC7075888; doi:10.1038/s41467-020-15220-0)
Supplement: Supplementary file 1 — Supplementary Information [file 41467_2020_15220_MOESM1_ESM.pdf]

Supplementary Information to the manuscript titled

“Dissecting the early steps of MLL induced leukaemogenic transformation using a mouse model of AML”

Basilico *et al*.

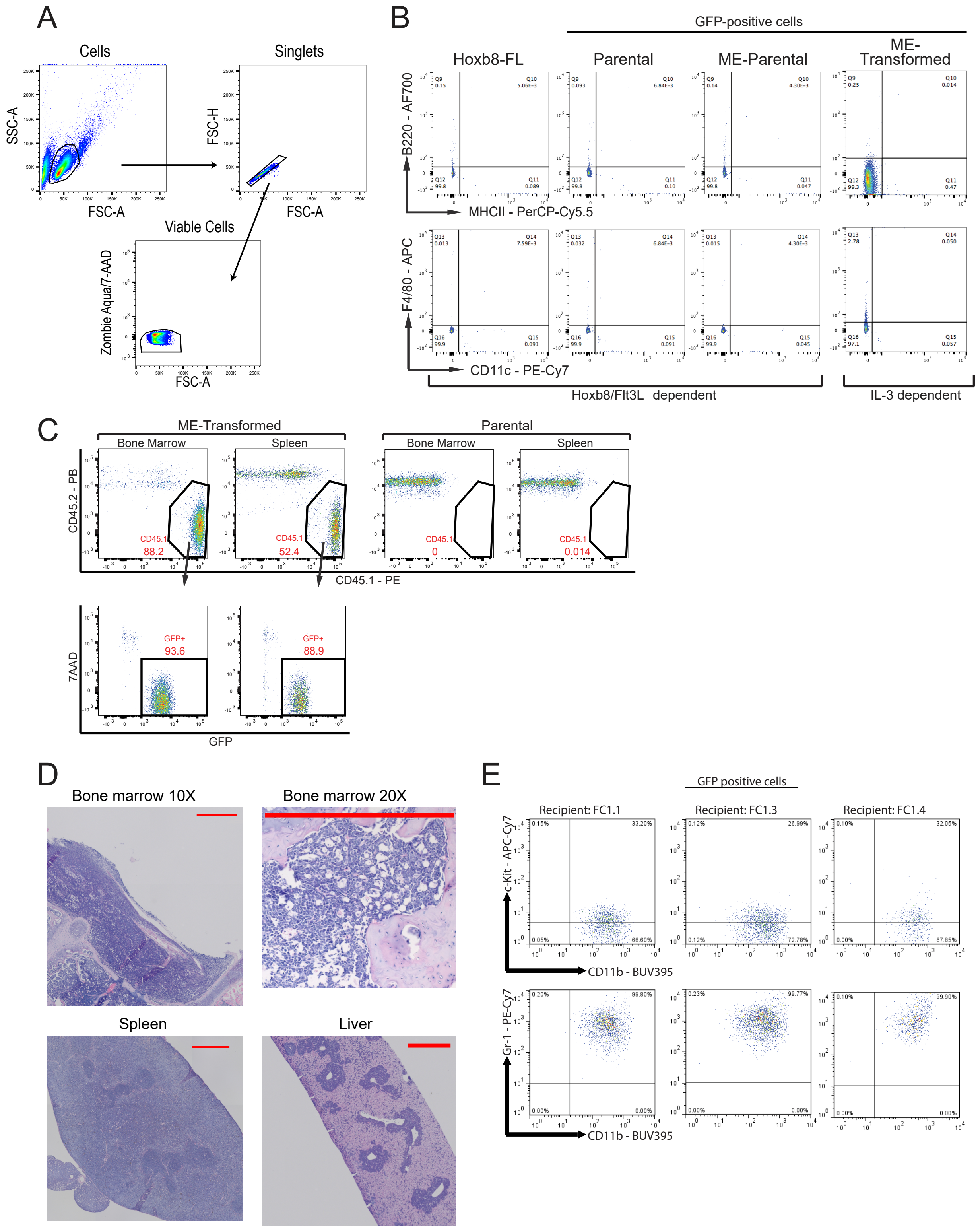

Supplementary Figure 1. Characterisation of ME-Transformed cells.
(A) Gating strategy for all populations analysed in all subsequent flow cytometry analyses. **(B)** Phenotypic characterisation of ME-transformed cells cultured in the presence of IL-3 in comparison to cells untransduced (Hoxb8-FL) and transduced with either empty vector or MLL-ENL (Parental and ME-Parental, respectively) cultured in the presence of Flt3L and β-estradiol. Expression of antigen presenting cell and macrophage markers is shown. **(C)** Representative flow-cytometry plots showing presence of CD45.1+ cells and their relative positivity for GFP in the bone marrow and spleen of mice transplanted with either ME-Transformed (n=5) or Parental cells (n=5). **(D)** Representative histology sections of bone marrow, spleen and liver of deceased mice transplanted with ME-Transformed cells. Size bars indicate 500µm. Similar results were found in 2 more mice. **(E)** Characterisation by flow cytometry of GFP-positive cells recovered from 3 different deceased mice transplanted with ME-Transformed cells.

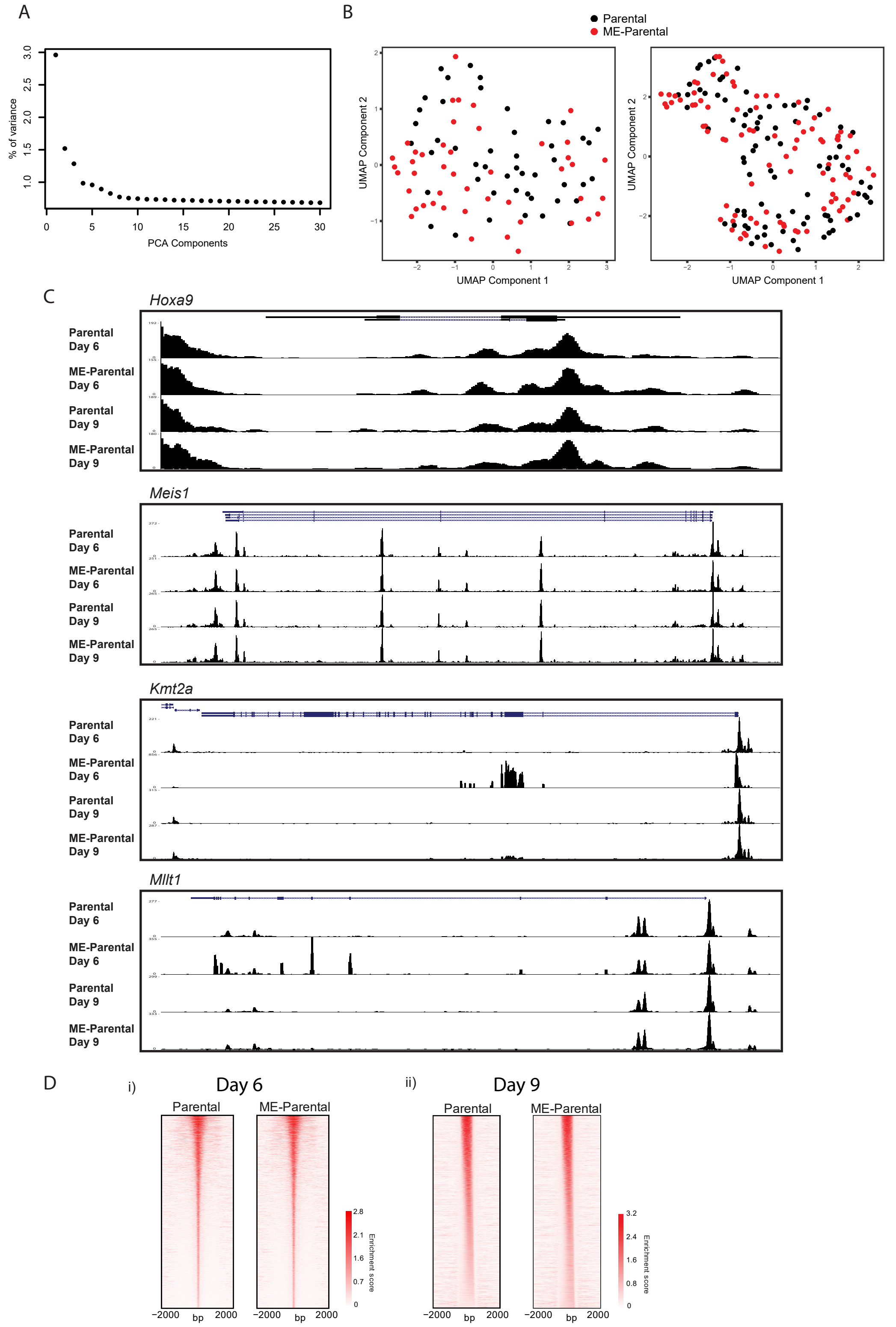

Supplementary Figure 2. ME-Parental cells have a similar transcriptome and chromatin profile to Parental cells.

(A) Scree plot showing the amount of variance explained by each component of the PCA in Figure 2B, which shows that Parental and ME-Parental cells have a similar transcriptome. **(B)** UMAP visualisations of single cell transcriptomes of Parental and ME-Parental cells obtained after 9 days of transduction and cultured in the presence of Flt3L and β-estradiol. 2 independent experiments are shown. **(C)** ATAC-Seq tracks for Parental and ME-Parental cells cultured for 6 and 9 days in the presence of Flt3L and β-estradiol. Snapshots for *loci* known to be important for MLL leukaemias (*Hoxa9* and *Meis1*) and for the *loci* involved in the translocation (*Kmt2a* and *Mllt1*) are shown. **(D)** NGS plots of ATAC-seq peaks obtained after either 6 days (i) or 9 days (ii) of transduction and cultured in the presence of Flt3L and β-estradiol for Parental and ME-Parental cells. Peaks are ranked from high to low using their coverage in Parental samples. Each row represents one peak. Peaks are aligned at the centre of the region extended to ± 2000bp. Colour scheme indicates the enrichment score (log2 scale).

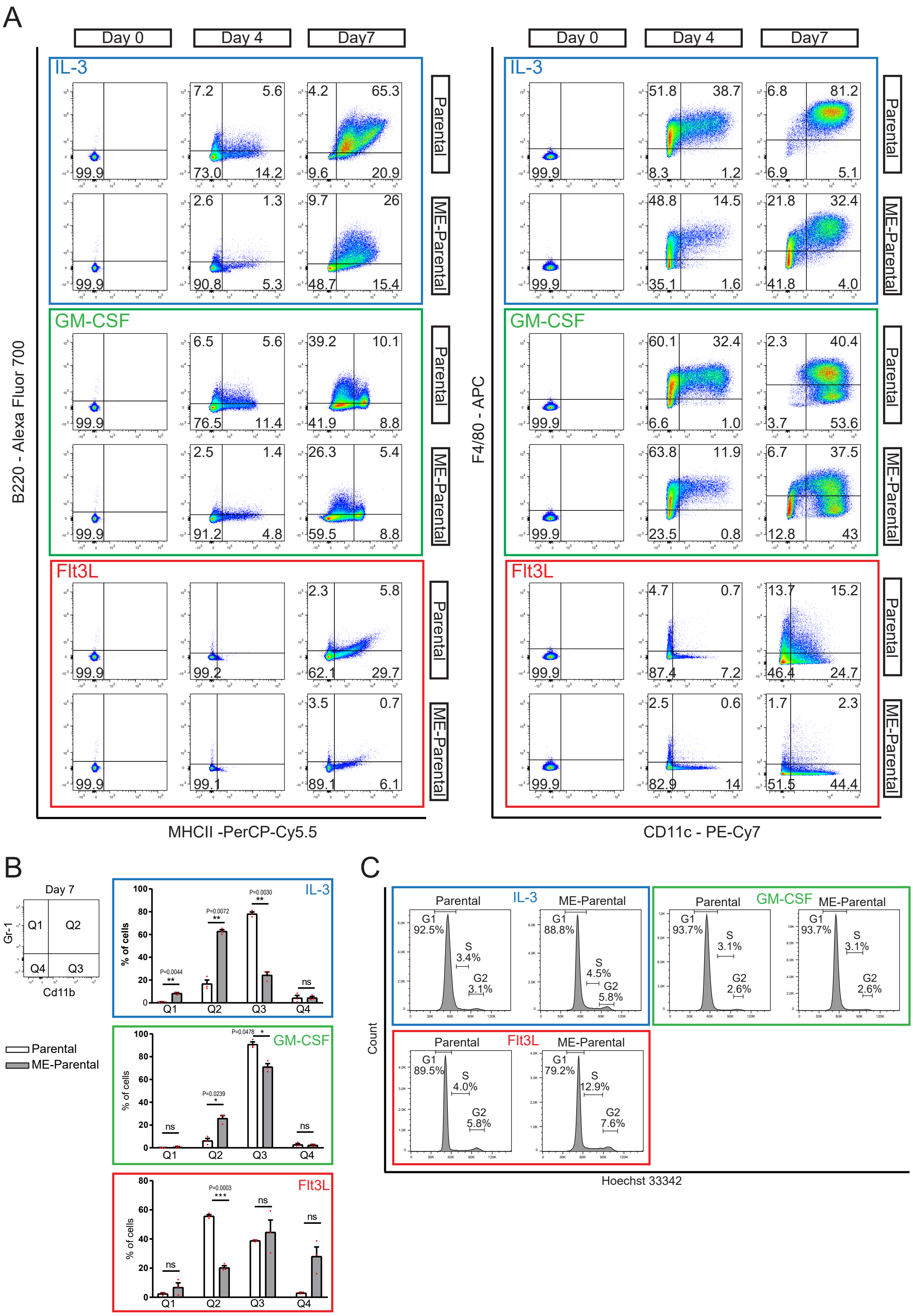

Supplementary Figure 3. Phenotypic and cell cycle effects of MLL-ENL fusion gene expression.
(A) Phenotypic analysis by flow cytometry of Parental and ME-Parental cells after differentiation for 4 and 7 days in the presence of either IL-3, GM-CSF or Flt3L. Day 0 represents Parental and ME-Parental cells before treatment (cultured in Flt3L and β-estradiol). Mean values are shown. N=3 biologically independent experiments. **(B)** Analysis of the phenotype obtained at day 7 of differentiation of Parental and ME-Parental cells in either IL-3, GM-CSF or Flt3L. Mean and SEM values for each of the four gates defined in the plots Cd11b against Gr-1 in Figure 3B are shown. Statistics were determined by two-tailed paired t-test. P-values are shown, also denoted as *p<0.05; **p<0.01; ***p<0.001. N=3 biologically independent experiments. **(C)** Representative cell cycle plots obtained by flow cytometry of Parental and ME-Parental cells after 7 days of differentiation in either IL-3, GM-CSF or Flt3L. Mean values are shown. N=4 biologically independent experiments. Source data are provided as a Source Data file.

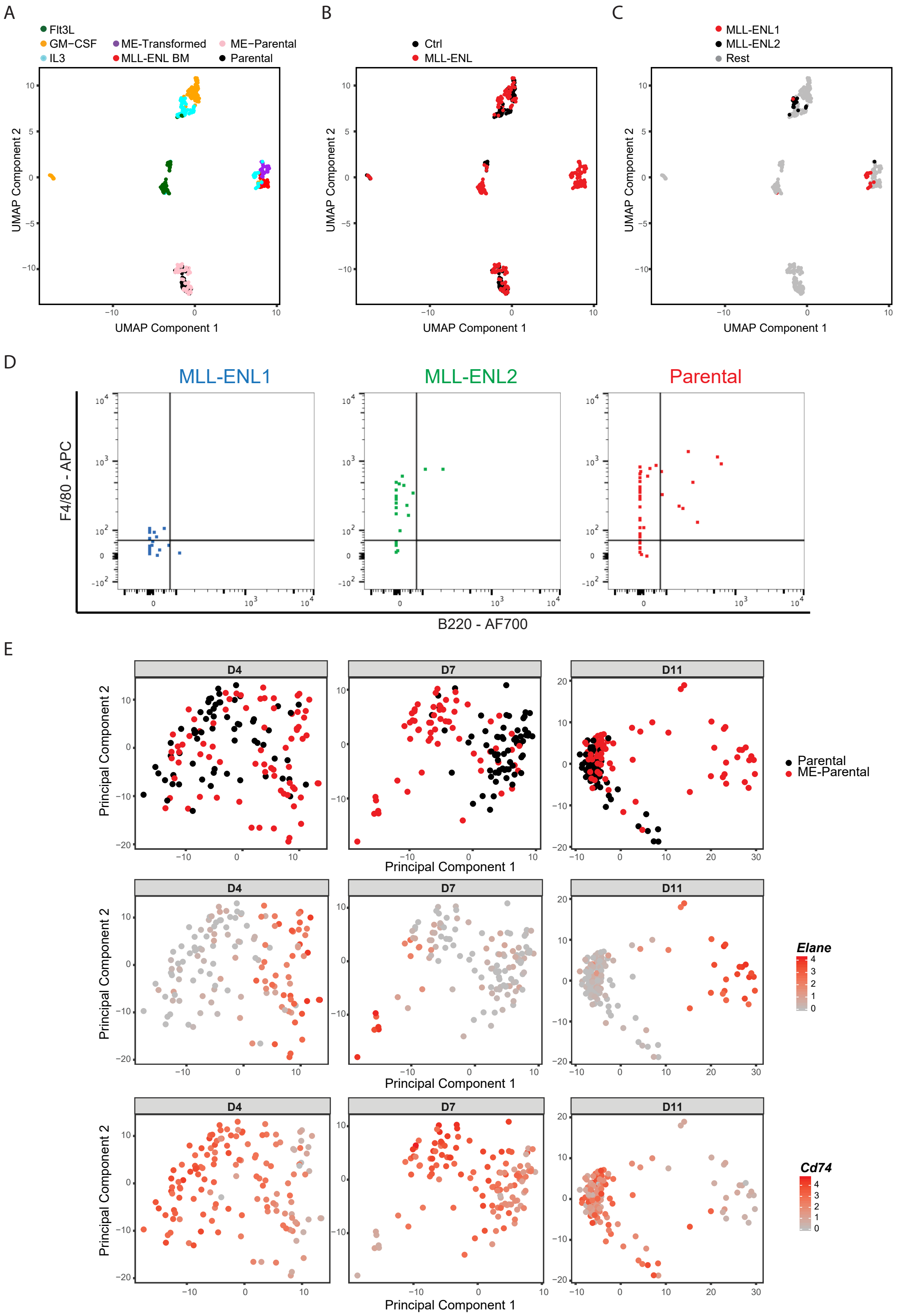

Supplementary Figure 4. Characterisation of MLL-ENL1 population

(A, B, C) UMAP representation of the transcriptomes obtained for Parental and ME-Parental cultured in the presence of either Flt3L and β-estradiol or myeloid cytokines (IL-3 or GM-CSF or Flt3L) for 7 days. For comparison, transcriptomes of ME-Transformed and MLL-ENL BM cells were projected in the same multidimensional space (see Supplementary Methods). Cells were coloured by either their identity (A), or if they have been transduced with a control or MLL-ENL virus (B), or if they belonged to MLL-ENL1, MLL-ENL2 cells or the rest as defined in Figure 4B (C). **(D)** Expression levels of B220 and F4/80 for MLL-ENL1, MLL-ENL2 and Parental cells obtained by index sort. **(E)** PCA visualisation of transcriptomes obtained for Parental and ME-Parental cells differentiated in IL-3 for 4, 7 and 11 days (left, middle and right columns, respectively). The expression of important genes to define MLL-ENL1 and MLL-ENL2 populations (*Elane* and *Cd74*) is shown for each cell. Colour scheme is based on log10 scale of normalised counts.

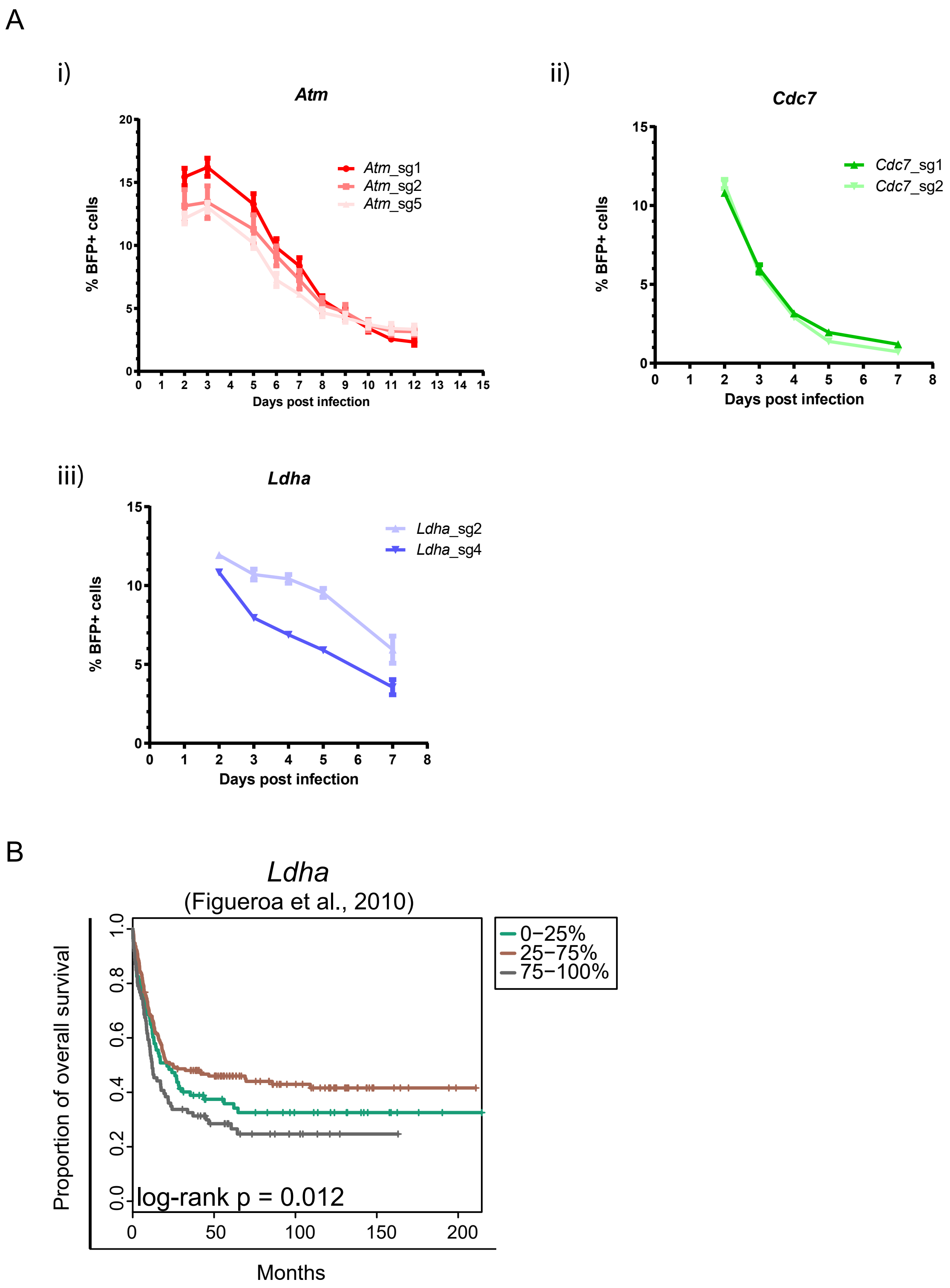

Supplementary Figure 5. Validation of targets obtained from genome-wide CRISPR-Cas9 screening. **(A)** Cells transduced with gRNAs against Atm (i), Cdc7 (ii) and Ldha (iii) were cultured together with untransduced cells. Transduced cells express the reporter gene BFP. Proportion of BFP-expressing cells was monitored by flow cytometry for up to 12 days post-transduction. Data are presented as mean +/- SEM. **(B)** AML patients' survival curves based on *Ldha* gene expression levels. Survival curves obtained via interrogation of Leukemia Gene Atlas. Green, orange and grey curves represent low (0-25%), medium (25-75%) and high (75-100%) expression levels of *Ldha* gene in AML patients, respectively. Statistics were determined by two-tailed log-rank Mantel-Cox test. Source data are provided as a Source Data file.

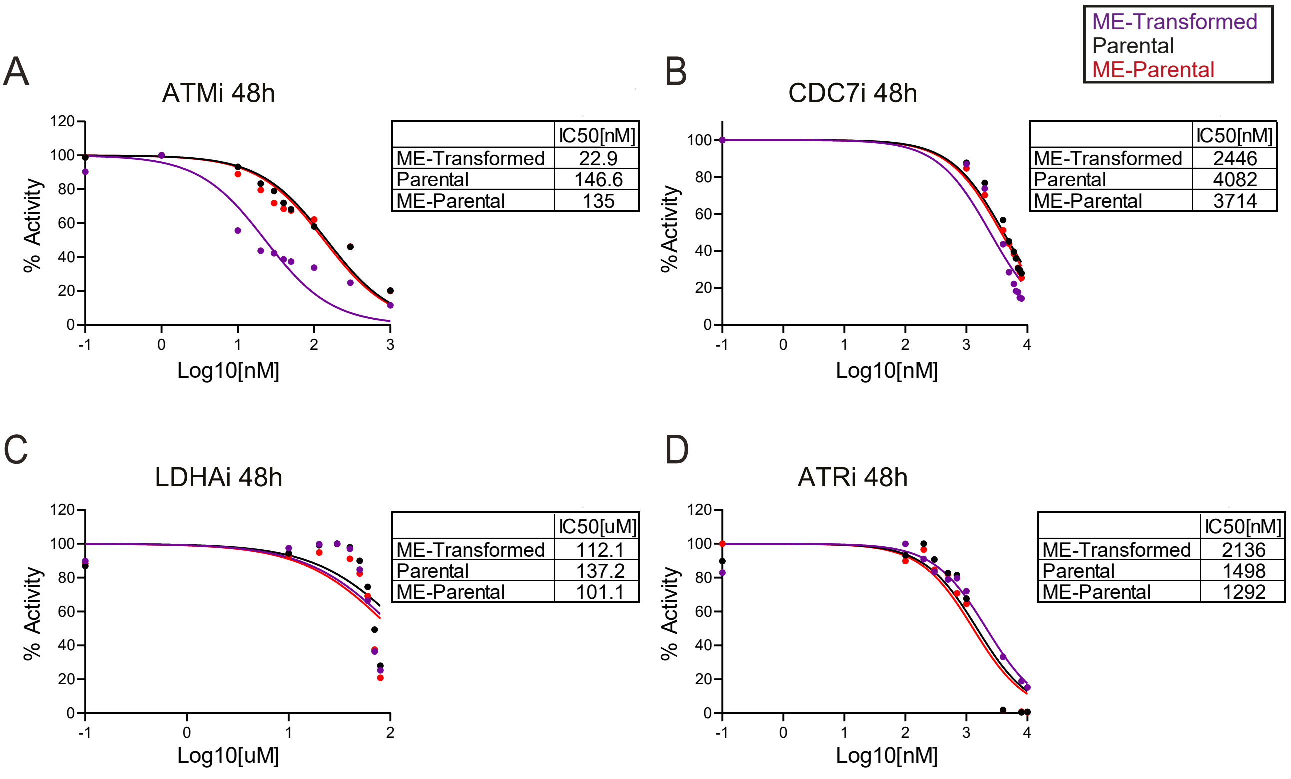

Supplementary Figure 6. ATMi, CDC7i, LDHAi and ATRi treatments.
(A), **(B)**, **(C)**, **(D)** Plots showing IC50 fitting curves and IC50 values at 48h post treatment. Data are shown as mean of two biological replicates; N=2. Source data are provided as a Source Data file.

Supplementary Methods

Retroviral and Lentiviral design and production

pMSCV-neo retroviral vector containing hMLL-ENL cDNA[1] (obtained from B. Huntly's laboratory) was digested with *ClaI* (NEB) and *XhoI* (NEB) to remove the neomycin resistance cassette. IRES-eGFP sequence was PCR amplified from pMSCV-PIG-IRES-eGFP vector (Addgene) using KAPA HiFi HotStart ReadyMix PCR Kit (KAPA BIOSYSTEMS) with primers designed to add *XhoI* and *ClaI* restriction sites. The IRES-eGFP fragment was then digested with *ClaI* and *XhoI* and ligated into pMSCV-MLL-ENL. The resulting vector was electroporated into DH10β *E. coli* and selected in ampicillin. Correct vector construction was confirmed by digestion with *BspeI* and via Sanger sequencing.

To produce retroviral particles, $2x10^6$ 293T cells were plated in 10cm plates 16-24 hours before transfection. Transfection reaction mixture consisted of 500μl of DMEM (no FBS or P/S), 5μg of pMSCV-MLL-ENL-IRES-eGFP retroviral vector, 5μg of pCL-Eco packaging vector and 30μl of TransIT-LT1 Transfection Reagent (Mirus Bio LLC). The mixture was incubated for 30 minutes at room temperature, then added dropwise to 293T-containing plates. 293T cells were cultured at 37℃ with 5% CO_2 for 24 hours and media was then replaced with 5ml of fresh media. Supernatant was collected 24 hours later, filtered through a 0.45μm filter and either used to infect cells or stored at -80℃.

Lentiviral particles were produced using the vectors pKLV2-EF1aBsd2ACas9-W, pMD2.G, VSV-G and pMCV-ΔR8.9 and TransIT-LT1 Transfection Reagent (Mirus Bio LLC) [2].

Retroviral/lentiviral transduction of Hoxb8-FL cell line

$1.5x10^6$ Hoxb8-FL cells in a 6-well plate were transduced in a final volume of 3 ml of media containing polybrene (Sigma) at a final concentration of 8μg/ml and 1ml of viral supernatant.

Plates were centrifuged at 800g for 90 minutes at 32℃ followed by incubation at 32℃ with 5% CO_2 for 90 minutes. Finally, 1.5ml of medium was carefully removed and replaced with 2.5ml of fresh Hoxb8-FL medium. Cells were incubated at 37℃ with 5% CO_2. 24 hours after transduction, 2ml of fresh medium was added to the cells.

Flow cytometry

Myeloid phenotypic characterization was performed using the following fluorophore-conjugated antibodies: Cd11b (BioLegend and BD Horizon, clone M1/70, dilution 1:100), c-Kit (BioLegend, clone 2B8, dilution 1:100), Gr-1 (BD Pharmingen, clone RB6-8C5, dilution 1:200), F4/80 (BioLegend, clone BM8, dilution 1:400), MHC Class II (Biolegend, clone M5/144.15.2, dilution 1:400), CD11c (BioLegend, clone N418, dilution 1:400), B220 (BioLegend, clone RA3-6B2, dilution 1:100) and Zombie Aqua Fixable Viability Kit (BioLegend, dilution 1:100) was used to exclude dead cells. Chimerism in mouse peripheral blood was assessed using the following antibodies: Cd45.1 (BioLegend, clone A20, dilution 1:100) and Cd45.2 (BioLegend, clone 104, dilution 1:100). Cells in mouse terminal tissues were characterised using the following antibodies: Cd45.1 (BioLegend, clone A20, dilution 1:100), Cd45.2 (BioLegend, clone 104, dilution 1:100), Gr-1 (BioLegend, clone RB6-8C5, dilution 1:100), c-Kit (BioLegend, clone 2B8, dilution 1:100) and Cd11b (BD Horizon, clone M1/70, dilution 1:100). 7-AAD (BD Pharmingen, dilution 1:60) was used to exclude dead cells.

Single cell RNA Sequencing

Cells were processed following the Smart-Seq2 protocol[3]. Briefly, cells were sorted in individual wells of 96-well plates that contained 2.3 µl of lysis buffer (0.2 % (v/v) Triton X-100 and 2 U/µl RNase inhibitor (Clontech)). Samples were maintained at -80C until processing. RT-PCR was performed in 10 µl containing 1 µM of oligo-dT, ERCCs (Ambion), 1mM dNTPs

(ThermoFisher), 5 mM DTT (Invitrogen), 1 M betaine (Sigma-Aldrich), 6 mM $MgCl_2$ (ThermoFisher), 1 µM TSO (Exiqon) and 100 U of SuperScript II (Invitrogen). The cDNA was preamplified in 25 µl using 12.5 µl of KAPA HiFi Hotstart Ready Mix (KAPA Biosystems) and 0.1 µM IS PCR primer. Amplified material was quantified using Scientific Quant-IT PicoGreen dsDNA Assay Kit (ThermoFisher) and ~150 ng of DNA was used to obtain Nextera XT libraries (Illumina). Libraries were then pooled and quantified using KAPA qPCR quantification kit (KAPA Biosystems). Pooled libraries were sequenced using a HiSeq 4000 sequencing machine, following a SE50 protocol.

We processed 48 Parental and 48 ME-Parental cells cultured in Flt3L and β-estradiol coupled to 48 Parental and 48 ME-Parental cells differentiated for 7 days in the presence of either Flt3L, IL-3 or GM-CSF. This experiment was repeated and in that occasion we processed 96 Parental and 96 ME-Parental cells cultured in Flt3L and β-estradiol coupled to 96 Parental and 96 ME-Parental cells differentiated for 4, 7 and 11 days in the presence of IL-3. We also processed 32 MLL-ENL BM cells and 32 ME-Transformed cells.

Sample analysis of scRNA-seq data was performed using R (https://www.r-project.org, v3.6.1). Raw sequence reads were mapped to *Mus musculus* genome GRCm38.81, the additional sequences of ERCCs and the construct used for transduction, using GSNAP[4] (v2015-09-29) with parameters -B 5 –n 1 –Q –N 1. Mapped reads were then related to gene features using script *htseq-count*[5] (version 0.6.0) with parameter –s no.

We then applied a quality control (QC) that involved: a) fraction of total reads associated to genes; b) number of reads mapping to nuclear genes; c) percentage of mapped reads mapping to mitochondrial genes; d) percentage of mapped reads mapping to ERCCs. The thresholds for these values were slightly adjusted for each of 3 different experimental batches.

Only cells passing quality control (QC) filters were kept for further analysis. A total of 1000 single cells passed QC distributed in 3 different batches as follows:

<table>
<tr><th>Condition</th><th>Treatment</th><th>Day</th><th>Batch 1</th><th>Batch 2</th><th>Batch 3</th></tr>
<tr><td rowspan="6">Parental</td><td>Untreated</td><td>Day 0</td><td></td><td>47</td><td>85</td></tr>
<tr><td rowspan="3">IL-3</td><td>Day 4</td><td></td><td></td><td>63</td></tr>
<tr><td>Day 7</td><td></td><td>37</td><td>66</td></tr>
<tr><td>Day 11</td><td></td><td></td><td>72</td></tr>
<tr><td>GM-CSF</td><td>Day 7</td><td></td><td>48</td><td></td></tr>
<tr><td>Flt3L</td><td>Day 7</td><td></td><td>16</td><td></td></tr>
<tr><td rowspan="6">ME-Parental</td><td>Untreated</td><td>Day 0</td><td>9</td><td>46</td><td>91</td></tr>
<tr><td rowspan="3">IL-3</td><td>Day 4</td><td></td><td></td><td>74</td></tr>
<tr><td>Day 7</td><td></td><td>42</td><td>62</td></tr>
<tr><td>Day 11</td><td></td><td></td><td>85</td></tr>
<tr><td>GM-CSF</td><td>Day 7</td><td></td><td>46</td><td></td></tr>
<tr><td>Flt3L</td><td>Day 7</td><td></td><td>47</td><td></td></tr>
<tr><td colspan="6">MLL-ENL BM 32</td></tr>
<tr><td colspan="6">ME-Transformed 32</td></tr>
</table>

On average, we obtained 1380562 reads mapped to nuclear genes, a median of 6539 total genes detected per cell and 4498 genes detected at more than 10 reads per million of total reads per cell.

Data normalization was done using *scran*[6] (v1.14.5) and estimation of highly variable genes (HVGs) was performed by fitting the squared coefficient of variation as a function of the mean normalised ERCC counts[7]. Normalised counts were transformed to $\log_{10}$ (counts+1) for dimensionality reduction and clustering.

Dimensionality reduction and batch integration

Principal component analysis (PCA) was done using the function *prcomp* in R. UMAP was calculated using the *umap* R package (v0.2.4.1) with default parameters based on the top 20 PCA components (seed value has been set to 0 to keep results consistent).

In Figure 2A, cells from two different batches were integrated using the fastMNN[8] function (v1.2.4) from the scran R bioconductor with k=5 based on the top 20 PCA components. Cells were integrated the same way in Figure 4E as for Figure 2A. In the case of Figure S4A, PCA was obtained for cells contained in Batch 2 alone and cells from Batch 1 were projected onto the Batch 2 PCA space. UMAP was then obtained using the top 20 PCA components.

Differential expression analysis

Pairwise differential expression analysis was performed using wald test in R bioconductor DESeq2[9] (v1.26.0). Key genes were selected from the DESeq2 results with a false discovery rate (FDR) (Benjamini-Hochberg method) lower than 0.1 and were used for the heat map plot using the function heatmap.2 in gplots R package (v3.0.1.2).

Gene annotation based on FastProject

Gene annotation was done using FastProject[10] (v1.1.4), which is a method that scores each cell against a gene signature. The final ranked scores were shown on PCA plots with red as higher association and green as lower association.

Projection on HSPC dataset

QC was ran on raw data matrix of both datasets (Dahlin et al., 2018[11] dataset (reference dataset) and scRNA-seq dataset) and only cells with more than 200 genes (with at least one count across all cells) were kept. Then cells were normalised to 10000 per cell. Following normalization, overlapping genes between the HVGs of both datasets were identified and normalization was done again with the overlapping genes. Then the datasets were log-transformed. Finally, top 50 PCs of Dahlin et al., 2018 dataset were calculated and scRNA-seq data was projected onto the PCA space of the reference dataset. Euclidian distance between projection data and

reference was calculated based on the top 50 PCA components and top 15 closest reference cells were selected for each projection cell. The SPRING plot in Figure 2C was coloured based on the number of reference cells selected. All the processing was performed using *scanpy*[12] (v1.4.5) in Python (v3.7).

ATAC-seq

Alignment and mapping

Reads were aligned to *Mus musculus* genome (GRCm38.81) using Bowtie2[13] (v2.2.5), then filtered for uniquely mappable reads. Reads were reduced to 1bp length based on the start coordinate.

Peak calling

Peak calling was run using F-Seq[14] (v3) with parameters: -t14 and -f1. A list of peaks was obtained for each of the two days (6 and 9) that resulted from the union of any peak called in either Parental or ME-Parental for each day and any overlapping peak regions were merged.

Visualisation tracks

The 1bp reads were counted in a sliding window of 75bp, sliding across the genome in 20bp bins, using the *bedmap* program from the BEDOPS toolkit[15] (v2.4.30). In-house scripts were then used to convert these counts into wiggle format for visualisation on the UCSC genome browser.

MA Plots

MA plots were generated using DESeq2[9] (v1.26.0).

Heat maps

Heat maps were generated using the NGS.plot package[16] (v2.61). Mapped reads (of ME-Parental and Parental cells) in BED format were converted to BAM using the bedToBam function from bedTools and sorted using the sort function from samTools. The R-package library for NGSplot was then run using parameters '-L 3000', '-GO none', and '-R bed', to generate heat maps using custom regions extended from the peak summit of +/- 2000bp. The regions had been pre-ranked based on enrichment values with the most enriched regions at the top using the genomeCoverageBed function from bedTools.

Pathway enrichment analysis

This analysis was performed with gene set enrichment analysis (GSEA) software (using the MSigDB[17] website http://software.broadinstitute.org/gsea/msigdb/annotate.jsp) and Enrichr[18] software (using the website http://amp.pharm.mssm.edu/Enrichr/). Results with adjusted p-value <0.05 (using Benjamini-Hochberg correction for multiple testing) were considered significant.

Identification of druggable genes

Drug Gene Interaction database (DGIdb)[19] (v3.02) was used to identify which of the genes obtained from the intersection of CRISPR-Cas9 and scRNA-seq data were potential druggable targets (http://dgidb.org/search_categories).

Leukaemia Gene Atlas (LGA)

LGA[20] (v2.1.0) was used to analyse which of the druggable genes identified by DGIdb had already been observed in AML patients. Survival rates were estimated for all datasets and

represented by Kaplan-Meier curves. Log-rank tests were performed to compare the survival curves and p-values <0.05 were considered significant.

Analysis of CRISPR-Cas9 data

The number of reads for each guide were counted with a script described in Tzelepis et al., 2016[2]. gRNA sequences were extracted by removing constant regions from each read and these were used to count the numbers of reads of each gRNA in the library. Depletion of guides and genes were analyzed using MAGeCK package[21] (v0.5.9) by comparing read counts from each mutagenized cell line with counts from matching plasmid as the initial population (control population not expressing Cas9).

Genetic Validation of Genome-Wide CRISPR-Cas9 Screening

Two or three gene-specific gRNAs were selected from the full library described in Tzelepis et al., 2016[2]. Individual gRNAs were cloned into expression vector pBA439 (Addgene, 85967) that contains blue fluorescent protein (BFP) as a reporter. Lentivirus was produced as described above. Cas9-expressing ME-Transformed cells were transduced with a lentivirus expressing a gene-specific gRNA, and the percentage of BFP-positive cells was measured via flow cytometry every 24-36 hours between 1 and 12 days post-transduction.

MTS Colorimetric assay

20000 cells were plated in individual wells of 96-well plates in a final volume of 100µl with increasing concentrations of inhibitor. No inhibitor and vehicle only conditions were also tested. All conditions were tested in duplicate. Cells were incubated at 37℃ with 5% CO2. When measured at 48h, 20 µl of combined MTS/PMS solution (Promega CellTiter 96® AQueous Non-Radioactive Cell Proliferation Assay, G5421) was added to each well.

Following incubation for 2 hours at 37℃ with 5% CO_2, absorbance at 490nm was measured on a SpectraMax M5e Microplate Reader (Molecular Devices LLC). When measured at 72h, cells were diluted 1:2 at 48h with fresh media (including inhibitor) resulting in a final volume of 200 µl. 72h after plating, 40 µl of combined MTS/PMS solution was added to each well of the plate and absorbance measured as previously described.

Data was imported into GraphPad software (v8.0) in XY format (X = drug concentration, Y = Aborbance). Concentrations were transformed into logarithms (log10). Transformed data was then normalized, subcolumns were averaged and means were normalized. 0% activity was defined as the absorbance of a well containing no cells and 100% was defined as the largest mean in each dataset. Values were then displayed as percentages. A curve with a fixed slope was fitted to the data from each cell line using nonlinear regression, calculating the IC50 for each curve.

Statistical analysis

Venn diagrams were generated using the website http://bioinfogp.cnb.csic.es/tools/venny/.

Dose-response curves were used to calculate IC50 via GraphPad software (v8.0).

Growth curves, survival curves and histograms were generated using GraphPad software (v8.0). Log-rank Mantel-Cox test was used for survival curve comparison considering significant the values with p-value <0.05. For comparison of two experimental groups, paired and unpaired *t*-tests were applied as indicated in the corresponding figure legend. Results are expressed as mean ± standard error of the mean (SEM). The statistical analysis was conducted at 95% confidence level. A p-value <0.05 was considered as statistically significant.

Exome Sequencing

gDNA extraction

Frozen stocks of spleen cells from 2 sacrificed mice (FC1.1 and FC1.3) (GFP+ cells <90% of total cells) were thawn, centrifuged at 300g for 5 minutes and washed once in 1xPBS. Cells were then stained with PE-AnnexinV (BD Pharmingen™) and incubated for 30 minutes at 4℃. Cells were washed once in 1X AnnexinV binding buffer and then resuspended in binding buffer and DAPI (1 µg/ml). Cell sorting was carried out on a Melody cell sorter (BD Biosciences). GFP+, AnnexinV-, DAPI- cells were sorted and expanded in M3434 Methocult (Stem Cell Technologies) for 3 weeks, replating every week. Frozen stock of bone marrow cells from 1 sacrifised mouse (FC1.5) (GFP+ cells > 90% of total cells) was thawn and immediately processed for DNA extraction, together with actively growing ME-Parental and Hoxb8-FL cells. DNA was extracted using DNeasy Blood & Tissue Kit (Qiagen). DNA was then treated with RNase A (Thermo Fischer) to remove contaminating RNA.

Exome capture, sequencing and analysis

Genomic DNA was captured using Agilent SureSelect XT Mouse All Exon baits. Whole-exome sequencing was performed using the Illumina HiSeq 4000 platform to generate 75bp paired-end reads. Sequencing reads were aligned to the reference genome GRCm38 using BWA mem (v1.14.9) [22] and PCR duplicates were flagged using 'bamstreamingmarkduplicates' in the 'biobambam2' package (v2.0.79)[23]. The mean sequencing depth was 193x (median 192x). Single nucleotide variants and indels were identified using 'cgpCaVEManWrapper' (v1.13.14)[24] and 'cgpPindel' (v3.3.0)[25], respectively, and default filters were applied. Single nucleotide variants were further filtered by removing calls within 5bp of an indel call. Indels in simple repeat region were removed, leaving a single indel event that was excluded after visual inspection. The Ensembl Variant Effect Predictor (v96)[26] was used to predict the effect

of variant changes on amino acids and protein sequences using gene models from Ensembl release 96. Variants from *Mllt1* and *Kmt2a* were excluded, as these were artefacts derived from the alignment of the human oncogene MLL-ENL (which was introduced into Hoxb8-FL cells) to the mouse reference genome.

Supplementary References

1. Horton, S. J. *et al.* Continuous MLL-ENL Expression Is Necessary to Establish a "Hox Code" and Maintain Immortalization of Hematopoietic Progenitor Cells. *Cancer Res* **65**, 9245–9252 (2005).
2. Tzelepis, K. *et al.* A CRISPR Dropout Screen Identifies Genetic Vulnerabilities and Therapeutic Targets in Acute Myeloid Leukemia. *Cell Reports* **17**, 1193–1205 (2016).
3. Picelli, S. *et al.* Full-length RNA-seq from single cells using Smart-seq2. *Nature Protocols* **9**, 171–181 (2014).
4. Wu, T. D., Reeder, J., Lawrence, M., Becker, G. & Brauer, M. J. GMAP and GSNAP for Genomic Sequence Alignment: Enhancements to Speed, Accuracy, and Functionality. in *Statistical Genomics: Methods and Protocols* (eds. Mathé, E. & Davis, S.) 283–334 (Springer New York, 2016). doi:10.1007/978-1-4939-3578-9_15.
5. Anders, S., Pyl, P. T. & Huber, W. HTSeq—a Python framework to work with high-throughput sequencing data. *Bioinformatics* **31**, 166–169 (2015).
6. Lun, A. T. L., McCarthy, D. J. & Marioni, J. C. A step-by-step workflow for low-level analysis of single-cell RNA-seq data with Bioconductor. *F1000Res* **5**, 2122 (2016).
7. Brennecke, P. *et al.* Accounting for technical noise in single-cell RNA-seq experiments. *Nature Methods* **10**, 1093–1095 (2013).

8. Haghverdi, L., Lun, A. T. L., Morgan, M. D. & Marioni, J. C. Batch effects in single-cell RNA-sequencing data are corrected by matching mutual nearest neighbors. *Nat Biotechnol* **36**, 421–427 (2018).
9. Love, M. I., Huber, W. & Anders, S. Moderated estimation of fold change and dispersion for RNA-seq data with DESeq2. *Genome Biology* **15**, 550 (2014).
10. DeTomaso, D. & Yosef, N. FastProject: a tool for low-dimensional analysis of single-cell RNA-Seq data. *BMC Bioinformatics* **17**, 315 (2016).
11. Dahlin, J. S. *et al.* A single-cell hematopoietic landscape resolves 8 lineage trajectories and defects in Kit mutant mice. *Blood* **131**, e1–e11 (2018).
12. Wolf, F. A., Angerer, P. & Theis, F. J. SCANPY: large-scale single-cell gene expression data analysis. *Genome Biol* **19**, 15 (2018).
13. Langmead, B. & Salzberg, S. L. Fast gapped-read alignment with Bowtie 2. *Nat Methods* **9**, 357–359 (2012).
14. Boyle, A. P., Guinney, J., Crawford, G. E. & Furey, T. S. F-Seq: a feature density estimator for high-throughput sequence tags. *Bioinformatics* **24**, 2537–2538 (2008).
15. Neph, S. *et al.* BEDOPS: high-performance genomic feature operations. *Bioinformatics* **28**, 1919–1920 (2012).
16. Shen, L., Shao, N., Liu, X. & Nestler, E. ngs.plot: Quick mining and visualization of next-generation sequencing data by integrating genomic databases. *BMC Genomics* **15**, 284 (2014).
17. Subramanian, A. *et al.* Gene set enrichment analysis: A knowledge-based approach for interpreting genome-wide expression profiles. *Proceedings of the National Academy of Sciences* **102**, 15545–15550 (2005).
18. Chen, E. Y. *et al.* Enrichr: interactive and collaborative HTML5 gene list enrichment analysis tool. *BMC Bioinformatics* **14**, 128 (2013).

19. Griffith, M. *et al.* DGIdb: mining the druggable genome. *Nat Methods* **10**, 1209–1210 (2013).
20. Hebestreit, K. *et al.* Leukemia Gene Atlas – A Public Platform for Integrative Exploration of Genome-Wide Molecular Data. *PLoS ONE* **7**, e39148 (2012).
21. Li, W. *et al.* MAGeCK enables robust identification of essential genes from genome-scale CRISPR/Cas9 knockout screens. 12 (2014).
22. Li, H. Aligning sequence reads, clone sequences and assembly contigs with BWA-MEM. *arXiv:1303.3997 [q-bio]* (2013).
23. Tischler, G. & Leonard, S. biobambam: tools for read pair collation based algorithms on BAM files. *Source Code Biol Med* **9**, 13 (2014).
24. Jones, D. *et al.* cgpCaVEManWrapper: Simple Execution of CaVEMan in Order to Detect Somatic Single Nucleotide Variants in NGS Data. *Curr Protoc Bioinformatics* **56**, 15.10.1-15.10.18 (2016).
25. Raine, K. M. *et al.* cgpPindel: Identifying Somatically Acquired Insertion and Deletion Events from Paired End Sequencing. *Curr Protoc Bioinformatics* **52**, 15.7.1–12 (2015).
26. McLaren, W. *et al.* The Ensembl Variant Effect Predictor. *Genome Biol.* **17**, 122 (2016).
