# Supplementary material for: Dissecting the early steps of MLL induced leukaemogenic transformation using a mouse model of AML
Source: Nat Commun. 2020 Mar 16;11:1407. doi: 10.1038/s41467-020-15220-0 (PMC7075888; doi:10.1038/s41467-020-15220-0)
Supplement: Supplementary file 3 — Description of Additional Supplementary Files [file 41467_2020_15220_MOESM3_ESM.pdf]

File Name: Supplementary Data 1

Description: **Exome Genome Sequencing Results.** This file contains relevant information about the mutations found in the different samples.

File Name: Supplementary Data 2

Description: **Differentially expressed genes in MLL-ENL1 and MLL-ENL2 populations.** Complete results of the different combinations of comparisons for the differentially expressed genes between MLL-ENL1, MLL-ENL2 and Parental cells obtained using DESeq2.

File Name: Supplementary Data 3

Description: **CRISPR-Cas9 screening Results.** Full results of CRISPR-Cas9 screening, including counts for each guide and gene for each time point of each experiment and cell type included in the analysis.

File Name: Supplementary Data 4

Description: **Comparison of Parental and ME-Parental results for CRISPR-Cas9 screening.** Summary of drop-out results for Parental and ME-Transformed cells. Also contains full results for Figure 5B.

File Name: Supplementary Data 5

Description: **Putative druggable genes specific for ME-Transformed cells.** Includes the full results for the comparison of specific ME-Transformed drop-outs and MLL-ENL1 specific upregulated genes (related to Figure 5C i)) and the full list of putative druggable genes and their categories (related to Figure 5C iii)).

File Name: Source Data

Description: **Source Data.** Contains the raw data underlying Figures 1B, 1D, 1F, 1G, 3B , 3C, 3D, 6A, 6B, 6C, 6D and Supplementary Figures 3A, 3B, 3C, 5, 6A, 6B, 6C and 6D.
