# Supplementary material for: Dissecting the early steps of MLL induced leukaemogenic transformation using a mouse model of AML
Source: Nat Commun. 2020 Mar 16;11:1407. doi: 10.1038/s41467-020-15220-0 (PMC7075888; doi:10.1038/s41467-020-15220-0)
Supplement: Supplementary file 9 — Reporting Summary [file 41467_2020_15220_MOESM9_ESM.pdf]

nature research

Corresponding author(s): Fernando J. Calero-Nieto
Berthold Göttgens

Last updated by author(s): Feb 10, 2020

Reporting Summary

Nature Research wishes to improve the reproducibility of the work that we publish. This form provides structure for consistency and transparency in reporting. For further information on Nature Research policies, see Authors & Referees and the Editorial Policy Checklist .

Statistics

For all statistical analyses, confirm that the following items are present in the figure legend, table legend, main text, or Methods section.

n/a	Confirmed	
☐	☒	The exact sample size (*n*) for each experimental group/condition, given as a discrete number and unit of measurement
☐	☒	A statement on whether measurements were taken from distinct samples or whether the same sample was measured repeatedly
☐	☒	The statistical test(s) used AND whether they are one- or two-sided<br>*Only common tests should be described solely by name; describe more complex techniques in the Methods section.*
☒	☐	A description of all covariates tested
☐	☒	A description of any assumptions or corrections, such as tests of normality and adjustment for multiple comparisons
☐	☒	A full description of the statistical parameters including central tendency (e.g. means) or other basic estimates (e.g. regression coefficient) AND variation (e.g. standard deviation) or associated estimates of uncertainty (e.g. confidence intervals)
☐	☒	For null hypothesis testing, the test statistic (e.g. *F*, *t*, *r*) with confidence intervals, effect sizes, degrees of freedom and *P* value noted<br>*Give P values as exact values whenever suitable.*
☒	☐	For Bayesian analysis, information on the choice of priors and Markov chain Monte Carlo settings
☒	☐	For hierarchical and complex designs, identification of the appropriate level for tests and full reporting of outcomes
☒	☐	Estimates of effect sizes (e.g. Cohen's *d*, Pearson's *r*), indicating how they were calculated

Our web collection on statistics for biologists contains articles on many of the points above.

Software and code

Policy information about availability of computer code

Data collection	Sequencing was performed in HiSeq2500 and HiSeq4000 and corresponding Illumina commercial software. FACS and flow cytometry data were collected using the BD FACSDIVA software.
Data analysis	Flow cytometry was analysed using FlowJo (v10.6.1). Graph Pad Prism v8 was used for Statistical analysis. For the analysis of scRNASeq and ATACSeq, R (v3.6.1), Python (v3.7), Scanpy (v1.4.5), scran (v1.14.5), gplots (v3.0.1.2), ggplot2 (v3.2.1), GSNAP (v2015-09-29), HTSeq-count (v0.6.0), Bowtie2 (v2.2.5), F-Seq (v3), BEDOPS toolkit (v2.4.30), bglab (v1.1), DESeq2 (v1.26.0), umap (v0.2.4.1), fastMNN (v1.2.4), FastProject (v1.1.4), NGS.plot (v2.61) were used. Also, Gene Set Enrichment Analysis (v7.0), Enrichr, Leukemia Gene Atlas (v2.1.0) and DGIdb (v3.0.2) were used for interpretation of the data. MAGeCK (v0.5.9) was used for the genome-wide CRISPR analysis. The analysis of the exome sequencing was performed with BWAmem (v.1.14.9), biobambam2 (v2.0.79), cgpCaVEManWrapper (v1.13.14), cgpPindel (v3.3.0) and Ensembl variant Effect Predictor (v96). The code has been deposited in GitHub (https://github.com/SharonWang/Basilico_NCpaper_Code).

For manuscripts utilizing custom algorithms or software that are central to the research but not yet described in published literature, software must be made available to editors/reviewers. We strongly encourage code deposition in a community repository (e.g. GitHub). See the Nature Research guidelines for submitting code & software for further information.

Data

Policy information about availability of data

All manuscripts must include a data availability statement. This statement should provide the following information, where applicable:
- Accession codes, unique identifiers, or web links for publicly available datasets
- A list of figures that have associated raw data
- A description of any restrictions on data availability

The genome-wide CRISPR screening reported in this paper has been deposited in the European Nucleotide Archive (http://www.ebi.ac.uk/ena) with numbers ERP118720 and ERS529672. scRNA-seq and ATAC-Seq data have been deposited in GEO (https://www.ncbi.nlm.nih.gov/geo/) with accession numbers GSE140807 and GSE141353, respectively. Data for exome sequencing have also been deposited in the European Nucleotide Archive with number ERP117027.

Field-specific reporting

Please select the one below that is the best fit for your research. If you are not sure, read the appropriate sections before making your selection.

[x] Life sciences [ ] Behavioural & social sciences [ ] Ecological, evolutionary & environmental sciences

For a reference copy of the document with all sections, see nature.com/documents/nr-reporting-summary-flat.pdf

Life sciences study design

All studies must disclose on these points even when the disclosure is negative.

Sample size	For experiments involving animals, sample size was estimated to give statistically significant differences when comparing between different conditions. For the size estimation in these experiments, two independent study groups, a binomial primary endpoint, 75% incidence in tested group, 0% incidence in the control group, a probability of type-I error of 5% and a probability of type-II error of 20% were assumed. No statistical method was used to predetermine sample size in the rest of experiments; 2-3 biological replicates with technical replicates were performed.
Data exclusions	For scRNA-Seq, samples were filtered through a quality control (QC) that involved: a) fraction of total reads associated to genes; b) number of reads mapping to nuclear genes; c) percentage of mapped reads mapping to mitochondrial genes; d) percentage of mapped reads mapping to ERCCs. Only cells passing quality control filters were kept for further analysis. No data was excluded for other experiments.
Replication	Reproducibility is addressed throughout the manuscript in the main text, methods, and figure legends.
Randomization	The experiments were not randomized. For animal experiments, animals were grouped by condition and mice in the 2 conditions were matched in age and sex. In scRNA-Seq and ATAC-Seq experiments, equal number of control and ME-transduced cells were processed simultaneously (and in the same plate for scRNA-Seq experiments). Differentiations and colorimetric assays of control and ME-transduced cells were performed in the same plate. Flow cytometry analysis of the differentiations were also performed in parallel.
Blinding	The investigators were not blinded to allocation during experiments and outcome assessment. Controls and ME-transduced samples were processed either simultaneously or in parallel in all experiments.

Reporting for specific materials, systems and methods

We require information from authors about some types of materials, experimental systems and methods used in many studies. Here, indicate whether each material, system or method listed is relevant to your study. If you are not sure if a list item applies to your research, read the appropriate section before selecting a response.

Materials & experimental systems

n/a	Involved in the study	
[ ]	[x]	Antibodies
[ ]	[x]	Eukaryotic cell lines
[x]	[ ]	Palaeontology
[ ]	[x]	Animals and other organisms
[x]	[ ]	Human research participants
[x]	[ ]	Clinical data

Methods

n/a	Involved in the study	
[x]	[ ]	ChIP-seq
[ ]	[x]	Flow cytometry
[x]	[ ]	MRI-based neuroimaging

Antibodies

Antibodies used	APC-Cy7 c-kit (clone 2B8 from BioLegend cat. 105826 lot. B254319) dilution 1:100<br>PE Gr-1 (clone RB6-8C5 from BD Pharmingen cat. 553128 lot. 7040522) dilution 1:200<br>BV711 CD11b (clone M1/70 from BioLegend cat. 01242 lot. B259991) dilution 1:100

	BUV395 CD11b (clone M1/70 from BD Horizon cat. 563553 lot8339988) dilution 1:100<br>APC F4/80 (clone BM8 from BioLegend cat. 123116 lot. B268075) dilution 1:400<br>PerCP-Cy5.5 MHC class II (clone M5/114.15.2 from BioLegend cat. 107625 lot. B269461) dilution 1:400<br>PE-Cy7 CD11c (clone N418 from BioLegend cat. 117318 lot. B222652) dilution 1:400<br>Alexa Fluor 700 B220 (clone RA3-6B2 from BioLegend cat. 103232 lot. B272460) dilution 1:100<br>CD16/CD32 (clone 93 from eBioscience cat. 14-0161-82 lot. 2071500) dilution 1:50<br>Zombie Aqua™ Fixable Viability Kit from BioLegend cat. 423101 lot. B279913 dilution 1:100<br>PE Cd45.1 (clone A20 from BioLegend cat. 110708 lot. B233347) dilution 1:100<br>PE-Cy7 Gr-1 (clone RB6-8C5 from BioLegend cat. 108416 lot. B248638) dilution 1:100<br>Pacific Blue Cd45.2 (clone 104 from BioLegend cat. 109820 lot. B279653) dilution 1:100<br>APC-CD45.2 (clone 104 from BioLegend cat. 109814 lot. B213058) dilution 1:100<br>BUV395 CD11b (clone M1/70 from BD Horizon cat. 563553 lot. 7306675) dilution 1:100<br>7-AAD from BD Pharmingen cat. 559925 lot. 8008681 dilution 1:60
Validation	Antibodies were validated as noted on manufacturer's website. Manufacturers tested their antibodies by immunofluorescent staining of mouse cells followed by flow cytometric analysis and comparison with isotype control.

Eukaryotic cell lines

Policy information about cell lines

Cell line source(s)	Hoxb8-FL cells were obtained from the laboratory of Dr. Hans Häcker (St. Jude Children's Research Hospital, Memphis, Tennessee, USA)
Authentication	Differentiation experiments in vitro, reconstitution experiments in vivo, flow cytometry and single cell RNASeq
Mycoplasma contamination	Cell lines tested negative for mycoplasma contamination
Commonly misidentified lines (See ICLAC register)	No commonly misidentified cell lines were used in the study

Animals and other organisms

Policy information about studies involving animals; ARRIVE guidelines recommended for reporting animal research

Laboratory animals	Six-week-old female C57BL/6J mice were bred and maintained at the University of Cambridge in microisolator cages and provided continuosly with sterile food, water and bedding.
Wild animals	The study did not involve wild animals.
Field-collected samples	The study did not involve field-collected samples.
Ethics oversight	All procedures were performed according to the United Kingdom Home Office regulations.

Note that full information on the approval of the study protocol must also be provided in the manuscript.

Flow Cytometry

Plots

Confirm that:

- [x] The axis labels state the marker and fluorochrome used (e.g. CD4-FITC).
- [x] The axis scales are clearly visible. Include numbers along axes only for bottom left plot of group (a 'group' is an analysis of identical markers).
- [x] All plots are contour plots with outliers or pseudocolor plots.
- [x] A numerical value for number of cells or percentage (with statistics) is provided.

Methodology

Sample preparation	In the case of Hoxb8-FL differentiations, cells were washed with PBS, incubated in FC-block (Biolegend) and stained with a mixture that contained antibodies against c-kit, Cd11b, Gr-1, Cd11c, B220, MHC Class II and F4/80. Terminal mouse tissues and peripheral blood analysis were performed in a similar way, using either Cd45.1 and Cd45.2 or c-kit, Cd11b and Gr-1 antibodies.
Instrument	Samples were analysed with either Influx cell sorter (BD Biosciences) or LSRFortessa (BD Biosciences).
Software	FACS and flow cytometry data were collected using the BD FACSDIVA software and analysed using FlowJo.

Cell population abundance	Abundances varied between different samples and conditions. Representative plots are shown in Figures and Supplementary figures in the manuscript.
Gating strategy	For FACS sorting, cells were stained with DAPI. Cells were firstly selected for FSC and SSC, followed by DAPI negative and GFP positive selection.<br>For flow cytometry analysis, cells were firstly selected for FSC and SSC followed by Zombie Aqua or DAPI negative selection.

☒ Tick this box to confirm that a figure exemplifying the gating strategy is provided in the Supplementary Information.
